# Pricing and reimbursement of novel oncology drugs in Sweden

**DOI:** 10.3402/jmahp.v2.25925

**Published:** 2014-12-09

**Authors:** Mattias Haglund, Eric Miller

**Affiliations:** 1 IIB Partner AB, Sweden; 2 Optio Biopharma Solutions, LLC, USA

Dear Editor,

We read with great interest the article by Cohen and Felix (1) concerning reimbursement restrictions on orphan drugs. We agree with their concerns that orphan drugs are often met with reimbursement restrictions, but data on the relationship between orphan status and pricing and reimbursement outcomes are scarce. Furthermore, regional disparities within Europe are likely to be significant, contributing to the previously reported heterogeneous uptake across different countries (2).

One could also argue that it is the increased focus on individual cost and the overall budget impact of orphan drugs, rather than lack of supporting clinical and cost-effectiveness data, that drives decreased reimbursement. Such budgetary and political pressure could counteract the original intent of legislators, which was to incentivize and increase interest in developing novel treatments for rare and severe diseases. It was as little as 10–15 years ago that the pharmaceutical industry was under fire for neglecting rare diseases and focusing only upon developing drugs for the highly prevalent (but commercially justifiable) disease markets. Thanks to progressive legislation and incentives, the tide has turned, and patients who suffer the unlucky fate of a rare disease now have hope of therapies like their fellow citizens with more prevalent illnesses.

In order to explore whether orphan drugs receive favorable or unfavorable pricing and reimbursement outcomes compared to non-orphan drugs within the same therapeutic class, we investigated publicly available reimbursement decisions by the Swedish pricing and reimbursement authority TLV, focusing particularly on oncology drugs. Sweden was chosen because 1) it has traditionally been considered a restrictive market for orphan drugs (3); and 2) the decisions of the TLV are made public immediately, along with a discussion of the arguments for and against reimbursement, lending itself well to qualitative analysis. The field of oncology was chosen because 1) a great number of therapies have recently been approved and launched, providing a number of observations amenable to analysis; and 2) the distribution of orphan and non-orphan drugs was expected to be fairly even, as opposed to other therapeutic areas known for a great number of orphan drugs (e.g. enzyme replacement therapy). We assessed treatment price, orphan status, as well as prevalence and information on cost per quality-adjusted life-year (QALY) and other published variables factoring in the decisions.

Out of 90 decisions reviewed, 26 concerned new oncology therapeutics. Of these, 17 concerned drugs with an orphan designation in the EU, and 9 did not. For the orphan drugs, 16 out of 17 received a favorable reimbursement decision, whereas 5 out of 9 non-orphan oncology drugs were rejected for reimbursement. The difference is statistically significant (*p*<0.01; chi square test).

This disparity in the rejection rate was observed despite the fact that drugs with an orphan designation were associated with a numerically higher price (average annual price ~60,000 EUR versus ~50,000 EUR; difference not statistically significant using the Mann–Whitney U-test), and in most cases they were associated with a cost per QALY that was significantly above the threshold for reimbursement traditionally applied by the TLV and other authorities. Thus, it appears that, at least in Sweden, an orphan designation may serve as a facilitator of reimbursement, associated with somewhat lower health technology assessment bars to clear than for non-orphan drugs.

In summary, while we agree that the concerns raised by Cohen and Felix are valid, relevant, and important, our findings in this limited data set indicate that, at least in Sweden, the intent of rare disease legislation is being realized. There is no evidence from this analysis of Swedish data that political cost containment pressure has caused orphan drugs to suffer undue reimbursement restrictions within their therapeutic category. In fact, oncology drugs with an orphan designation appear to fare somewhat better in the reimbursement process than oncology drugs without orphan designation.

Our analysis was an initial response with at best a limited answer to the questions posed by Cohen and Felix. Obviously, our findings have some inevitable limitations, and exploration of other therapeutic areas is warranted. For example, one could safely argue that the public does not differentiate between rare and more prevalent malignancies. A valid critique of our analysis is that ‘orphan’ anti-neoplastic agents may benefit from both rare-disease incentives and the political cover provided by high public awareness and support of cancer research as a whole. Another obvious limitation is that our conclusions may be valid for only a single EU country and may not be applicable in other countries. At the very least, we now have more data to aid in addressing an interesting and important question.

*Mattias Haglund* IIB Partner AB, Sweden Email: mh@iibpartner.com *Eric Miller* Optio Biopharma Solutions, LLC USA

## References

[1] Cohen J, Felix A (2014). Are payers treating orphan drugs differently?. J Mark Access Health Pol.

[2] Picavet E, Annemans L, Cleemput I, Cassiman D, Simoens S (2012). Market uptake of orphan drugs – A European analysis. J Clin Pharm Ther.

[3] Garau M, Mestre-Ferrandiz J (2009). Access mechanisms for orphan drugs: A comparative study of selected European countries. OHE Brief.

